# Study of the Effect of the Use of Asphalt Binders Modified with Polymer Fibres from End-of-Life Tyres (ELT) on the Mechanical Properties of Hot Mix Asphalt at Different Operating Temperatures

**DOI:** 10.3390/ma15217578

**Published:** 2022-10-28

**Authors:** Alejandra Calabi-Floody, Cristian Mignolet-Garrido, Gonzalo Valdes-Vidal

**Affiliations:** Department of Civil Engineering, Universidad de La Frontera, Temuco 4780000, Chile

**Keywords:** end-of-life tyres, polymer fibre, fibre-modified asphalt, mechanical properties, asphalt mixtures

## Abstract

Processing of end-of-life tyres (ELT) produces polymer fibres (PFELT) as a by-product. PFELT currently presents a challenge to the recycling industry, due to the increasing numbers of ELT and lack of alternatives for the re-use of this material. The object of this investigation was to propose an alternative for re-using PFELT, in order to improve the performance properties of hot mix asphalt (HMA). This study enabled us to understand the relation between the addition of polymer fibre to the aggregate-binder matrix of the HMA in depth, as well as its effects on the mechanical properties of the resulting asphalt mix. To do this, we first made a physical and chemical characterization of the PFELT (TGA, SEM, polarized light, and fluorescence microscopy), establishing a modification methodology using two asphalt binders (CA-24 and CA-14) and three PFELT contents (0.1%, 0.3%, and 0.5%). The HMA was designed using the Marshall method. The mechanical performance of the HMA was evaluated in a range of operating temperatures, from −10 °C to 50 °C, observing the following properties: (1) resistance to thermal cracking; (2) stiffness modulus; (3) indirect tensile strength; and (4) resistance to permanent deformation. The results show that the addition of 0.3% and 0.5% of PFELT to the asphalt binder significantly improved the mechanical performance properties of the mixes studied, with a greater effect at high operating temperatures; the resistance to permanent deformation increased by more than 30%.

## 1. Introduction

Over the years, the number of end-of-life tyres (ELT) has grown at an increasing rate, becoming a priority problem for many government entities. According to reports from 2018, around 20.6 million tons of ELT are disposed of around the world every year [1]. This is a worrying figure, considering the harmful effects of this waste on the environment and public health [2,3,4]. In this context, various ELT management systems have been developed to encourage the re-use of this waste, with the European union (EU) pioneering a new ELT treatment and management models [5] through regulatory systems, such as extended producer responsibility (EPR), the free market system, and the tax system [6,7]. According to the European Tyre and Rubber Manufacturers Association (ETRMA), in the period 2019–2020, around 3.2 million tons of ELT were collected and treated in Europe [8], through the implementation of different strategies, such as re-use (5–23%), recycling (3–15%), energy recovery (25–60%), and disposal in landfill (20–30%) [9]. Nevertheless, efforts have been inconsistent, and the final destination of a large part of the ELT generated by the European tyre industry is unknown. This material has a direct impact on the environment.

The principal components of ELT are: rubber (45–47%), steel wire (12–24%), and polymer fibre (1–10%) [10,11]. The composition of these components may vary, depending on the type of tyre processed. The greatest emphasis is placed on the re-use of rubber [12,13,14,15,16,17,18] and steel wire [10,19,20]. Nevertheless, the polymer fibre obtained from ELT (PFELT) represents a challenge for the recycling industry; a small amount is used as combustion material in cement works, and the rest is disposed of in landfills [21,22]. The fibres obtained from ELT consist mainly of polyester polymers and Nylon 6 or Nylon 6.6 [21,22].

Various studies have been published evaluating the use of polymers as modifiers in asphalt binders, making these less susceptible to temperature, thus improving the mechanical properties of hot mix asphalt (HMA). Polymers can be grouped into three categories: thermoplastic elastomers, plastomers, and reactive polymers [23]. Each polymer in these three groups has a specific effect on the performance of asphalt binders. Styrene-based thermoplastic elastomers (SBS, SEBS, SIS) are used to obtain good elastic properties in a modified asphalt binder, while plastomers (vinyl acetate, polyester, and polypropylene) and reactive polymers are added to improve stiffness and reduce permanent deformation [24]. One of the polymers most frequently used today by the industry to modify asphalt binder is SBS (styrene butadiene styrene); however, its use implies an increase of around 20% in the cost of the final product (modified asphalt), as compared to a conventional asphalt binder [25]. Studies also exist evaluating the effects of a wide range of polymer fibres, both natural (flax, linen, coconut, and hemp) and synthetic (carbon, glass, polyester, nylon, and aramid), on the mechanical properties of asphalt mixes [26], finding that they generally achieve an improvement in resistance to permanent deformation and fatigue. Mohammed et al. [27] studied the rheological properties of asphalt binder modified with cellulose and glass fibres (0.5%, 1.0%, and 2.0% by volume of asphalt), finding an increase in the consistency, temperature of the softening point, and viscosity of the various samples evaluated with fibre. Based on these results, Mohammed et al. [28] studied the relative performance of asphalt mixes modified with the same cellulose and glass fibres; however, in this study, they studied the performance with the addition of the steel wire recovered from tyres. The authors determined by microscope analysis that the steel wire formed a sort of three-dimensional network in the asphalt mix matrix, with uniform distribution and orientation. In mechanical trials, the mixes with fibre presented better performance in indirect tensile strength, resistance to thermal cracking, fatigue damage, and moisture damage. Klinsky et al. [29] evaluated the performance characteristics of HMA mixes modified with polypropylene and aramid fibres (0.05% by weight of the mix), finding that polypropylene fibres provided some adherence by acting as a dispersant, while the three-dimensional network of aramid fibres strengthens the mix. In the performance properties, the authors found that the mixes modified with fibre presented better behaviour in the stiffness modulus at 25 °C, resistance to permanent deformation, and resistance to fatigue damage at moderate to low deformation levels. Kim et al. [30] studied the mechanical properties of asphalt mixes modified with polypropylene, polyester, nylon, and carbon fibres (0.5% and 1.0% by volume of the mix). The authors found that polyester and nylon fibres presented improved behaviour for the following parameters: Marshall stability, indirect tensile strength, susceptibility to moisture, dynamic stability, and low-temperature bending, especially if the fibre content is increased from 0.5% to 1.0%; the exception was the case of dynamic stability, which presented no significant increase. In another study, Noorvand et al. [31] investigated the distribution and effect on mechanical properties of aramid fibres added to asphalt mixes. The authors found that the more dispersed and distributed fibres have a positive effect on the general performance of asphalt mixes. They also showed that the orientation of the fibres was a decisive factor in the fatigue and permanent deformation behaviour.

According to these studies, the addition of different types of fibre is shown to improve the mechanical properties of asphalt binders and asphalt mixes. Since the principal component of PFELT is polyester, it is estimated that the use of asphalt binder modified with PFELT in HMA mixes will strengthen the aggregate-binder matrix of the asphalt mix, producing similar results to those observed in the studies mentioned above. The object of this investigation was, therefore, to study an alternative for re-using PFELT to improve the performance properties of hot mix asphalt (HMA). This object was addressed through an in-depth investigation of the effects of the incorporation of asphalt binders modified with PFELT on the mechanical properties of hot mix asphalt. The study explored a wide range of operating temperatures of the asphalt mixes and evaluated the mechanical properties at high, intermediate, and low temperatures, in the range 50 °C to −10 °C. The study included two asphalt binders (CA-24 and CA-14) and three contents of added PFELT

## 2. Materials and Methods

### 2.1. Raw Materials

#### 2.1.1. Asphalt Binder Modifier Additive

In this study, we used a polymer fibre obtained from ELT recycling, denoted in the present investigation as PFELT. This material consists of translucid filaments and rubber particles remaining from the ELT separation process (approximately 60% weight) [10,32]. The PFELT used in this study was sieved (mesh size 2.36 mm) to separate out most of the rubber waste remaining after ELT recycling. The main components of the PFELT were polyester (99.6%) [21] and polyamide 6.6 [18]. The basic characteristics of the PFELT are shown in Table 1.

#### 2.1.2. Asphalt Binder

Two conventional asphalt binders were used: CA-24 and CA-14 classified, according to the Chilean standard [34]. The properties of the asphalt binders are shown in Table 2.

#### 2.1.3. Stone Aggregates

The aggregates used were of riverbed origin and complied with the Chilean standard for use in surface courses [35]. The aggregates were mainly composed of particles of dolomite, basalt, dacites, andesites, rhyolites, sandstone, quartz, and quartzite. The properties of the aggregates used are shown in Table 3.

### 2.2. Modification of Asphalt Binder

**Figure 1:**
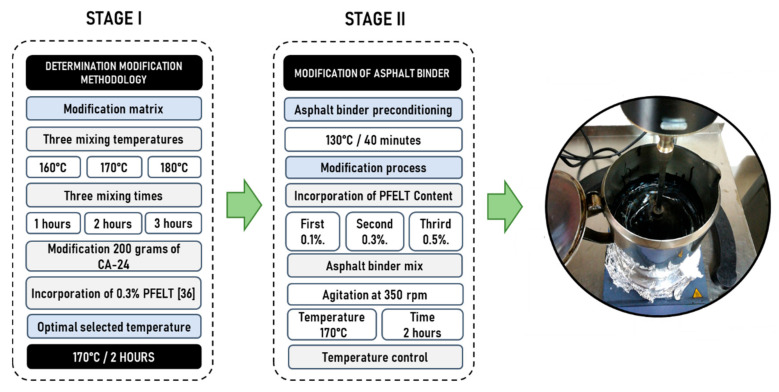
Modification process of the asphalt binder with different contents of PFELT.

A time/temperature modification matrix was applied to determine the most suitable modification procedure to obtain homogeneous samples, without affecting the ageing of the asphalt binder. Samples of 200 gr of CA-24 asphalt binder were trialled. Three temperatures (160, 170, and 180 °C), and three mixing times (1 h, 2 h and 4 h) were evaluated, all using the same proportion of PFELT (0.3% by weight of asphalt binder [36]). The best modification condition was used to modify the two asphalt binders evaluated (CA-24 and CA-14). In order to evaluate the effects on the mechanical properties of HMA, three PFELT contents (0.1%, 0.3%, and 0.5% by weight of asphalt binder) were used in this study. The modification process is shown in Figure 1.

### 2.3. Mix Design

**Figure 2:**
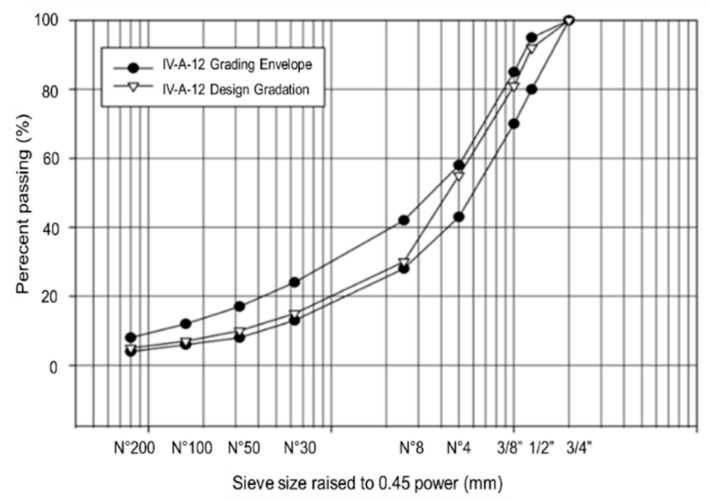
Aggregate gradation of the IV-A-12 reference asphalt mixture.

A semi-dense granulometry was used: type IV-A-12, according to the Chilean standard (Figure 2). The maximum asphalt content was determined using the Marshall method. The optimum asphalt content for the reference mixes was 5.2% by weight of aggregates. This dosification was maintained for the asphalt mixes manufactured with the asphalt binders modified with PFELT.

### 2.4. Testing Methods

**Figure 3:**
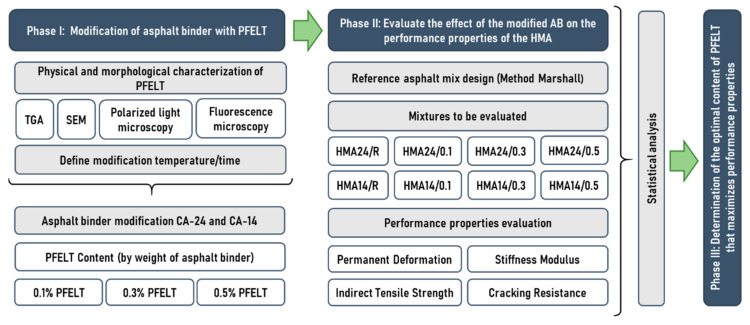
Research methodology.

To evaluate the effect of the PFELT on the mechanical properties of the HMA, a three-phase sequential testing methodology was applied. The methodology is shown in Figure 3.

#### 2.4.1. Phase I

In this phase, the physical and morphological characterisation of the PFELT was established, allowing us to determine the optimum modification methodology through a time/temperature evaluation matrix. The TGA/DSC thermogravimetric curve was obtained by thermogravimetric analysis (TGA), in order to determine the thermal decomposition point of PFELT. A simultaneous thermal analyser model STA6000 (Perkin Elmer) was used. The surface characteristics and morphology of the PFELT were analysed by microscopy tests (SEM and polarized light). SEM and polarized light microscopy images were obtained at a scale of 500 and 300 μm, respectively. In addition, fluorescence microscopy was used to analyse the distribution and dispersion of the PFELT, evaluated through the time/temperature modification matrix proposed in this study (Section 2.2 Modification of Asphalt Binder). This procedure shows typical morphologies of the additive mixed in a black asphalt binder matrix, allowing observation of the level of additive integration resulting from the modification conditions [37]. The test was performed using an Olympus FV1000, Olympus, evaluating the excitation/emission spectra at 3 wavelengths: 405/450, 488/530, and 633/750 nm, with a magnification of 20× and a depth of 30 μm.

Based on these analyses, the optimum modification time and temperature were determined; these were then used in the modification of two conventional asphalt binders (CA-24 and CA-14) with 0.1%, 0.3%, and 0.5% of PFELT (by weight of asphalt binder).

#### 2.4.2. Phase II

In this phase, the mechanical properties of the asphalt mixes manufactured with PFELT-modified asphalt binders were assessed. The binders complied with the Marshall criteria. In the rest of this paper, the mixes manufactured with CA-24 and CA-14, modified with 0.1%, 0.3%, and 0.5% of PFELT, will be designated as HMA24/01, HMA24/03, HMA24/05 and HMA14/01, HMA14/03, and HMA14/05, respectively. The reference mixes manufactured with CA-24 and CA-14 will be designated as HMA24/R and HMA14/R, respectively. These mixes were evaluated at high, intermediate, and low temperatures, applying tests to observe the effects on the resistance to rutting at high temperature, stiffness modulus, indirect tensile strength at intermediate temperature, and cracking resistance at low temperature.

Resistance to rutting was evaluated by the Hamburg wheel tracking test (HWTT), in accordance with the AASHTO T 324 standard [38]. This method allows the degree of permanent deformation and the stripping potential of asphalt mixes to be determined. The load applied was 705 ± 4.5 N for 10,000 cycles under the action of water, at a temperature of 50 ± 0.5 °C. Four specimens were manufactured and tested for each type of asphalt mix evaluated, using the Superpave compaction method, until 7 ± 1% air void was reached, as established in European regulations.

The stiffness modulus was determined according to European standard EN 12697-26 Annex C [39] at 25 °C. Three cylindrical Marshall specimens per type of asphalt mixture studied were manufactured (compacted by 75 blows per face), with a total of 12 specimens tested.

The indirect tensile strength (*ITS*) was studied, following the EN 12697-23 protocol [40]. This procedure consists in subjecting cylindrical test specimens to a compression load across the vertical diameter of the test specimen, at a velocity of 50 mm/min and temperature 25 °C. The *ITS* parameter is the maximum traction force calculated from the maximum load applied when fracture occurred, according to Equation (1). Three cylindrical Marshall specimens per type of asphalt mixture studied were manufactured (compacted by 75 blows per face), with a total of 12 specimens tested.

(1) $$ITS=\frac{2\cdot P}{\begin{array}{c} \pi \cdot D\cdot H \end{array}}\cdot 100$$

where *ITS* is the indirect tensile strength (kPa), *P* is the maximum load applied (kN), *D* is the diameter of the test specimen (mm), and *H* is the height of the test specimen.

**Figure 4:**
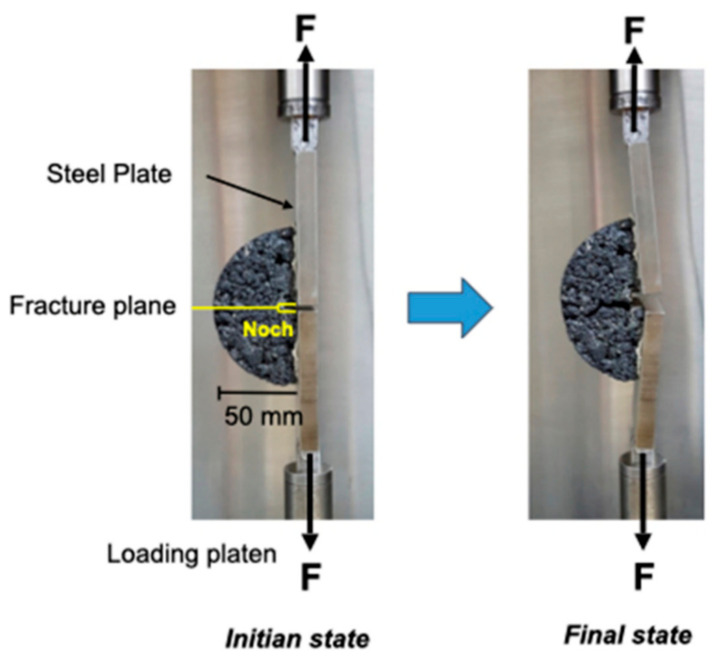
Cracking processes of the specimen by Fenix test.

The low temperature cracking resistance was determined by the Fenix test (Figure 4). This test simulates a Mode I fracture, the principal cracking mechanism of asphalt mix [41]. The test procedure followed the protocol described in Spanish standard NLT-383/20 [42]. It consists in applying a traction force to half a cylindrical test specimen at a constant displacement velocity of 1 mm/min, at a specific temperature. In this investigation, the test was carried out at −10, 0, and 10 °C. The main parameters of the Fenix Test, i.e., the maximum traction force (Fmax) and the displacement at 50% of the post-peak traction force (d50PM), can be determined from the load-displacement curve recorded during the test (Figure 4). Three cylindrical Marshall specimens per type of asphalt mixture studied were manufactured (compacted by 75 blows per face), with a total of 12 specimens tested.

The results obtained in the evaluation of the performance properties were verified by statistical analysis. The assumptions of normality and homoscedasticity of the dataset were verified by the Shapiro–Wilk and Levene’s tests, respectively. The type of test (parametric or non-parametric) to be carried out was determined on the basis of these analyses. The fit of the data to a normal distribution was evaluated by linear regression analysis, a parametric test. Pearson’s correlation coefficient was used to study the relation between the study variables. The data which did not fit a normal distribution were evaluated by non-parametric tests, considering that all the test specimens evaluated were mutually related. The non-parametric tests used were the Friedman and Wilcoxon signed-rank tests. All the test results were analysed with a 95% confidence interval.

#### 2.4.3. Phase III

Analysis of the results obtained in the experimental tests, and of the statistical analyses, allowed us to determine the optimum addition of asphalt binder modifier to maximise the performance properties of the asphalt mixes evaluated, namely: (1) resistance to permanent deformation; (2) stiffness modulus; (3) indirect tensile strength; and (4) cracking resistance.

## 3. Results and Discussion

This section reports the results obtained in the tests carried out on the asphalt mixes manufactured with asphalt binder modified by addition of three different percentages of PFELT.

### 3.1. Characterisation of the Addition of Asphalt Binder Modifier

The object of Phase 1 was to characterise the PFELT to determine the time/temperature modification matrix. This information allowed proper integration of the PFELT in the asphalt binder.

#### Thermogravimetric and Microscope Analysis of the PFELT

**Figure 5:**
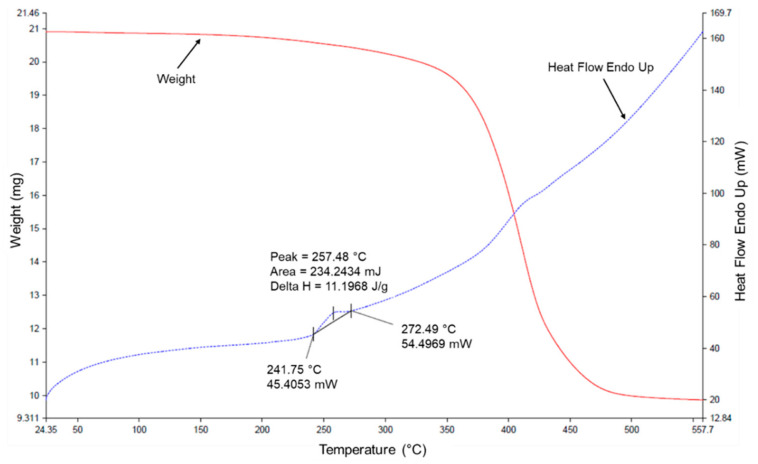
Thermogravimetric curves TGA of PFELT.

The thermogravimetric analysis (TGA) used to characterize the physical and chemical properties of the PFELT is shown in Figure 5. The results of the TGA curves show that the melting point of the PFELT evaluated in this study was 241.75 °C, while, at temperatures between 300 and 500 °C, the material suffered thermal decomposition by devolatilization (loss of volatiles and structural changes). These characteristics agree with those reported in other studies in which fibres obtained from ELT have been evaluated, of melting point approximately 259 °C and temperature for the start of decomposition (devolatilization) around 347 °C [18,43,44]. From this, it may be concluded that the PFELT cannot melt at the temperatures evaluated in the modification matrix proposed for this study.

**Figure 6:**
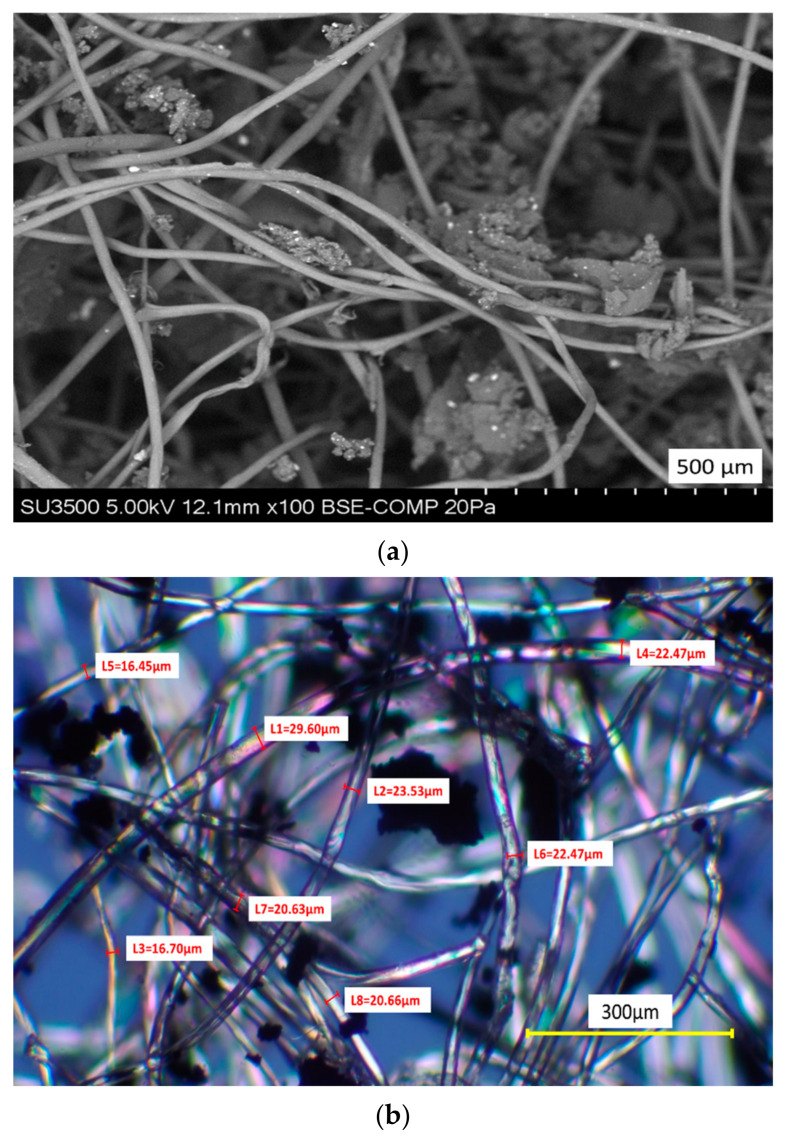
Morphological characterization of the PFELT: (**a**) SEM; (**b**) polarized light microscope.

The microscope analyses (SEM and polarization) used to characterize the morphology of the PFELT are shown in Figure 6. The sweep electron microscope (SEM) and polarized light microscope results show that the polymer fibre studied consists of filaments of mean diameter 22.5 μm, with particles of rubber waste from ELT recycling adhering to the fibres; this agrees with other studies [10,32].

**Figure 7:**
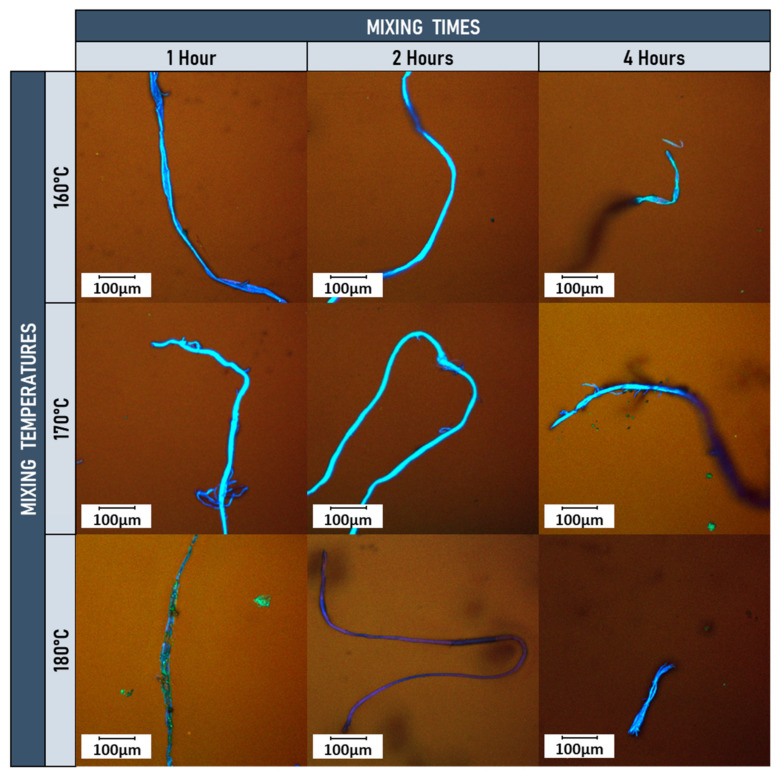
Fluorescence images for PFELT temperature/time modification matrix.

The fluorescence microscope analyses of the samples evaluated using the time/temperature modification matrix proposed in the study are shown in Figure 7. These results show that at temperatures of 160 and 170 °C, for periods of 1 and 2 h, the PFELT presents no structural alterations attributable to the modification process. At higher temperatures (180 °C) and longer modification periods (4 h), some degree of degradation is observed in the PFELT, related with the higher exposure temperature and/or longer mixing periods. Total disintegration of the PFELT is not observed however, which agrees with the results obtained in the TGA analyses; this indicates that the fibre evaluated only starts to decompose above 300 °C. Based on these analyses, we determined an optimum process temperature of 170 °C and mixing time of 2 h; this combination gives homogeneous, uniform samples and is intended to avoid accelerated ageing of the asphalt binder. This agrees with Fang et al. (2015), who indicated that the higher the modification temperatures, the more the asphalt binder is affected by oxidation (ageing), thus altering its physico-chemical properties [45].

### 3.2. Analysis of the Effect of the Asphalt Binder Modified with PFELT on the Mechanical Performance of the HMA

The object of Phase 2 was to analyse whether the modification of asphalt binder by the addition of PFELT has any effect on performance at high, intermediate, and low temperatures in the different asphalt mixes evaluated. In the rest of this article, we will refer to asphalt binder modified with PFELT as AB-PFELT.

#### 3.2.1. Evaluation of the Effects of PFELT on the Mechanical Properties of HMA at High Temperature

**Figure 8:**
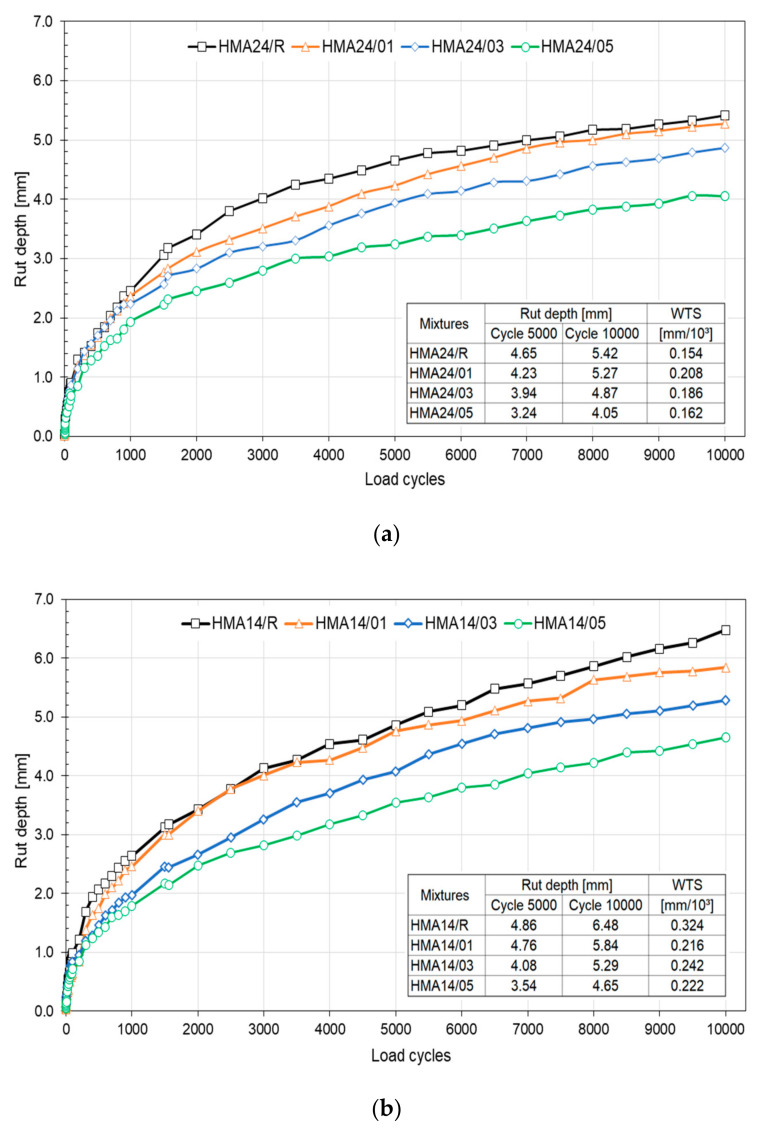
Hamburg wheel tracking test results at 50 °C: (**a**) HMA24; (**b**) HMA14.

Figure 8 shows the values for the mean rut depth (RD) under the action of water at 50 °C obtained in the Hamburg wheel tracking test. We observed that the reference mixes HMA24/R and HMA14/R presented lower resistance to permanent deformation. In the case of the HMA24 mixes, we observed that the rut depth in HMA24/R was up to 34% greater than in HMA24/05. A similar trend is observed for the HMA14 mixes: the rut depth observed in the HMA14/R mix was 39% greater than that observed in HMA14/05. In both mixes, a trend was observed for the RD parameter in the test asphalt binder to diminish with increased PFELT content, and in comparison with the reference mixes (Figure 8a,b). This improvement in the mechanical performance of HMA mixes in their resistance to permanent deformation agrees with the study of Eskandarsefat et al. (2019) [46], who indicated that the addition of fibre strengthens the matrix of the asphalt binder, thus generating an improvement in properties, such as viscosity and resistance to rutting at high temperatures. In relation to workability, it is important to note that HMA/24 mixtures with the incorporation of AB-PFELT require more compaction energy as the PFELT content in the AB increases. The HMA24/01, HMA24/03, and HMA24/05 mixes required −3.5%, 5.8% and 50.7% more compaction energy respectively than the reference mixture HMA24/R. However, the same trend was not observed for the HMA/14 mixture. The HMA14/01, HMA14/03, and HMA14/05 mixes recorded a compaction energy difference of 24.9%, −2.0%, and 9.2%, respectively, compared to the reference mixture HMA14/R.

Turning to the deformation gradient (WTS), between cycles 5000 and 10,000, we observed that the HMA14 mixes manufactured with AB-PFELT presented a lower deformation gradient that the HMA14/R mix. In contrast, the HMA24 mixes with AB-PFELT presented higher values in the deformation gradient, even though they showed better performance in resistance to permanent deformation, agreeing with the studies of Mahrez et al. (2005) and Klinsky et al. (2018) [29,47]. It should be noted that the WTS gradient diminishes after cycle 7000; the progression of the damage, therefore, decreases also, and it is to be expected that this trend will be maintained. Furthermore, we observed that none of the mixes evaluated presented loss of adhesion between the stone aggregates and asphalt binder (stripping), showing that the addition of AB-PFELT does not affect the resistance of HMA mixes to moisture damage.

The linear regression analysis shows that the data obtained from the HWTT fit a distribution, which complies with normality and homoscedasticity, with a *p*-value above the level of significance (0.05) in the Shapiro–Wilk (*p* > 0.100) and Levene’s (*p* = 0.613) tests. According to the statistical analysis, evidence exists, with 95% confidence, that the addition of AB-PFELT and type of asphalt binder evaluated produce significant changes (*p* < 0.05) in the RD parameter. This effect is greater when the mixes include AB-PFELT, indicating that the F value is sufficiently high, in order to allow for the mean performance in this property of the mixes to differ statistically.

#### 3.2.2. Evaluation of the Effects of PFELT on the Mechanical Properties of HMA at Intermediate Temperature

**Figure 9:**
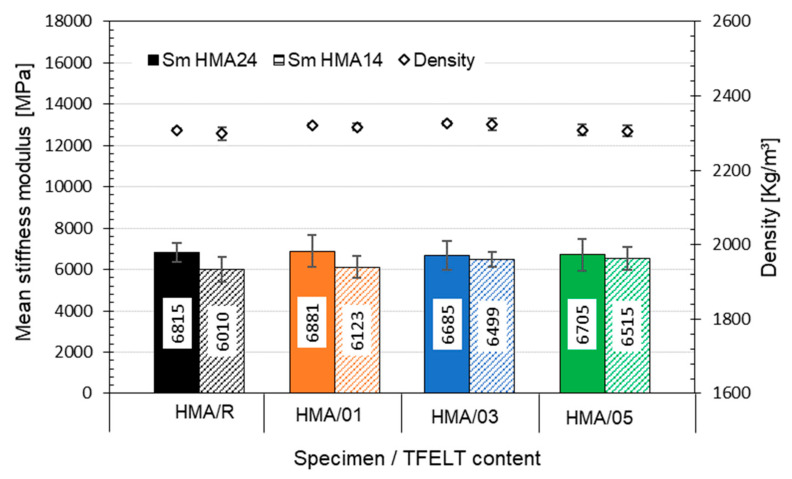
Hamburg results of stiffness modulus at 25 °C for all the mixes studied.

Figure 9 shows the mean values obtained for the stiffness modulus at 25 °C. No significant difference was observed between the stiffness modulus of the HMA24 mixes with addition of AB-PFELT and that of the HMA24/R mix (*p* > 0.05). On the other hand, in the HMA14 mixes with the addition of AB-PFELT, the mean value of the stiffness modulus tends to rise with the increase in the amount of PFELT added to the asphalt binder; however, no significant difference was observed, in comparison with the HMA14/R mix (*p* > 0.05). This shows that this property was unaffected in the mixes manufactured with AB-PFELT, since they presented a similar behaviour to that observed for the HMA/R. Figure 9 shows that very similar density values were recorded for all the mixes evaluated, thus indicating that the addition of AB-PFELT does not affect the density of HMA.

**Figure 10:**
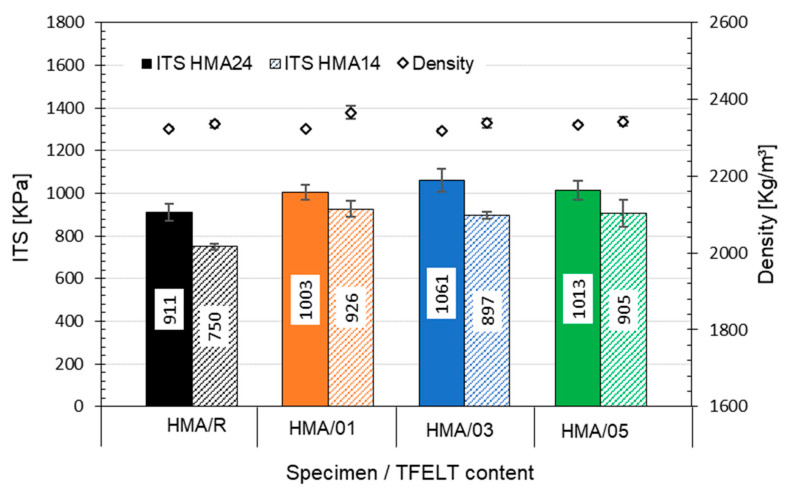
Indirect tensile strength test results at 25 °C for all the mixes studied.

Measurement of the indirect tensile strength at 25 °C (Figure 10) shows that the HMA mixes, with addition of AB-PFELT, have greater tensile strength. In the HMA24 mixes, a significant increase (*p* = 0.042 < 0.05) is observed in the tensile strength of the HMA24/01, HMA24/03 and HMA24/05 mixes over that of the HMA24/R mix, with *ITS* values that are 9%, 14%, and 10% higher, respectively. A similar pattern is observed for the HMA14 mixes, where significant increases (*p* = 0.037 < 0.05) are observed for HMA14/01, HMA14/03, and HMA14/05 over HMA14/R, of 19%, 16%, and 17%, respectively. This finding agrees with the studies of Kim et al. (2018), Shukla et al. (2013) and Kumar et al. (2009) [30,48,49]; these studies used synthetic and mineral fibres in asphalt mixes, such as polypropylene, polyester, nylon, and glass, finding an increase of up to 12% in the tensile strength, compared to the conventional mixes evaluated. This increase in the *ITS* value of HMA mixes with addition of AB-PFELT may be due to the good adhesion between the PFELT and the asphalt binder, thus allowing the PFELT to absorb part of the tensile stress. This would translate into an improvement in the performance in this property of the asphalt binders, as indicated by Chen and Lin (2005) [50]; this benefit would be passed on to the mechanical performance of the mix, thus improving the tensile strength. On the other hand, the densities of the mixes with AB-PFELT remains similar to those of the HMA/R mixes, thus confirming our observations in the evaluation of the stiffness modulus.

#### 3.2.3. Evaluation the Effects of PFELT on the Mechanical Properties of HMA at Low Temperature

**Figure 11:**
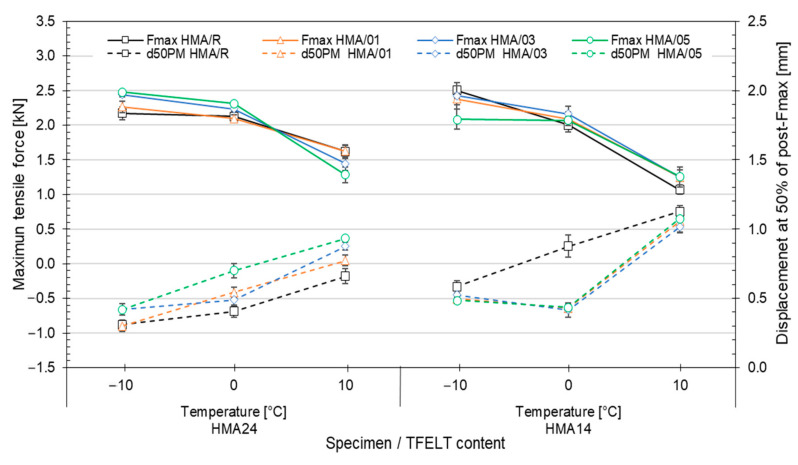
Fenix test results at −10 °C, 0 °C and 10 °C.

The Fenix parameters related with the maximum traction force (Fmax) and bending capacity (d50PM) of the mixes evaluated are shown in Figure 11. For the HMA24 mixes, we observed that at −10 °C and 0 °C the mixes with AB-PFELT presented better resistance to thermal cracking, with increased tensile strength and deformation capacity. The best performances were recorded for HMA24/03 and HMA24/05, with significantly higher values (*p* < 0.05) in the Fmax parameter at both temperatures (5–12% and 9–14%, respectively) than were found for the HMA24/R mix. At 10 °C, the differences observed in the Fmax parameter showed no statistically significant difference (*p* > 0.05), so there is no evidence of an important influence of the AB-PFELT on the behaviour of this property (R^2^ = 0.9089). However, the Pearson’s correlation coefficient shows a moderate positive correlation (r = 0.53), indicating that the higher the content of added PFELT in the asphalt binder, the greater the tensile strength of the mixes, but that the effect is moderate. The bending capacity after the start of fracture (d50PM) presented significant improvement at all the temperatures evaluated and for all the HMA24 mixes with addition of AB-PFELT (*p* = 0.018 <0.05), showing greater capacity of the mixes to admit deformation at a lower temperature; this indicates that the addition of AB-PFELT gave better resistance to thermal cracking (R^2^ = 0.9582).

The tensile strength (Fmax) of the HMA14 mixes with addition of AB-PFELT showed no significant effects at the temperatures evaluated (*p* > 0.05), as compared to the HMA14/R mix (R^2^ = 0.9651), indicating a weak negative Pearson’s correlation coefficient (r = −0.35); from this, it may be estimated that the higher the percentage addition of PFELT to the asphalt binder, the lower the tensile strength of the mixes, but that the effect is weak. Furthermore, all the HMA14 mixes with addition of AB-PFELT at the temperatures evaluated presented lower bending capacity, with a significant diminution (*p* = 0.04 < 0.05) in the d50PM parameter, compared to the HMA14/R mix (R^2^ = 0.9971). This effect indicates that the addition of PFELT to the asphalt binder made no difference at low temperatures in the HMA14 mixes, and that the mixes had a faster fracture velocity.

### 3.3. Determination of the Optimum AB-PFELT Content

**Figure 12:**
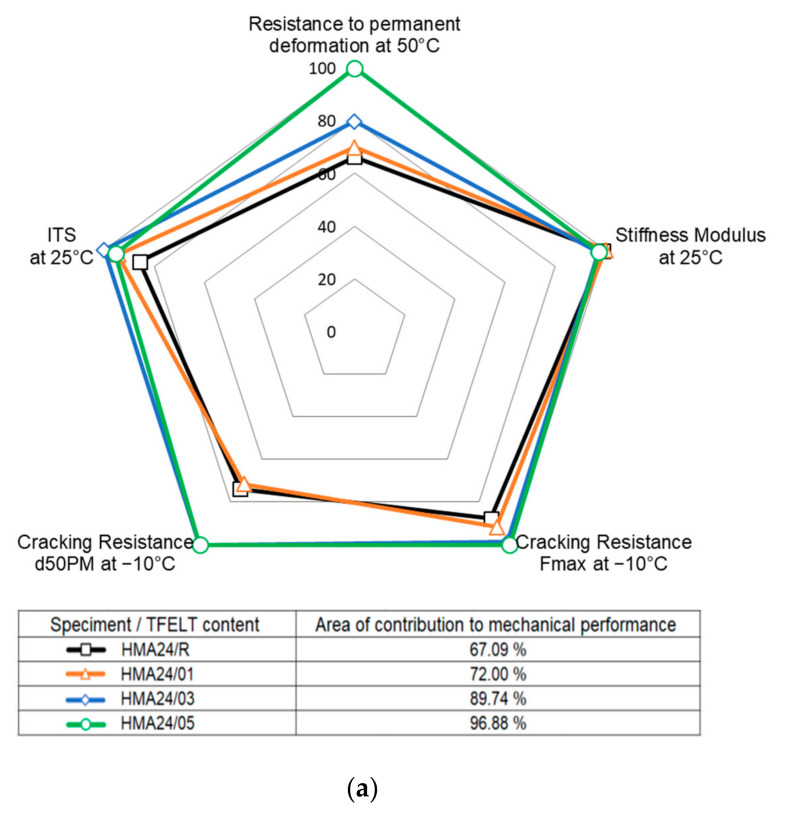

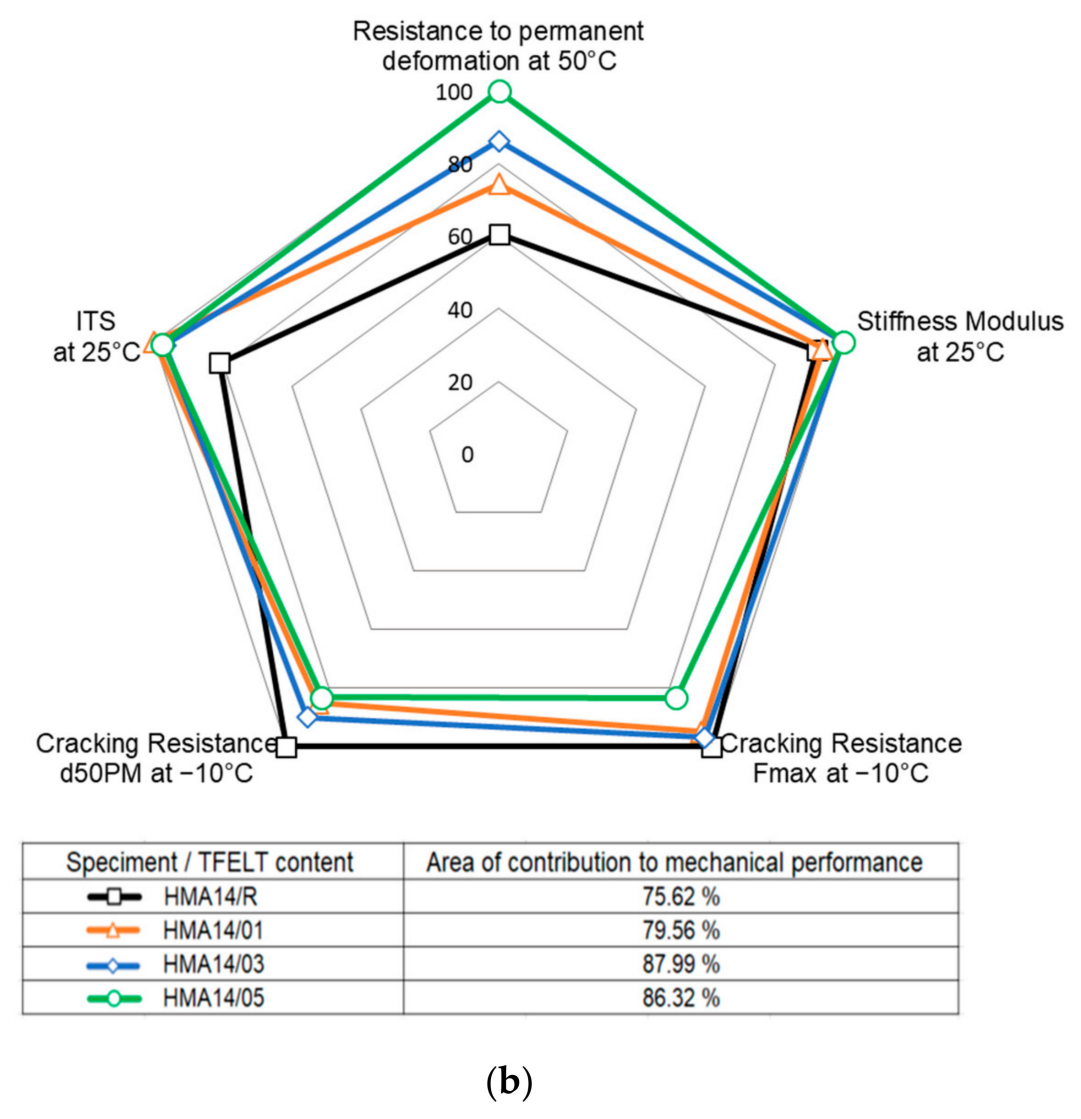
Radar graphs for the contribution to mechanical performance of the HMA mixes manufactured with AB-PFELT: (**a**) HMA24; (**b**) HMA14.

The object of Phase 3 was to determine the optimum content of PFELT addition in the asphalt binder, in order to maximise the performance properties of the asphalt mixes evaluated. Figure 12 shows the overall performance of the asphalt mixes evaluated, relating their resistance to permanent deformation, stiffness modulus, maximum tensile strength, and resistance to thermal cracking. The effects of the addition of AB-PFELT are expressed in percentage terms; the value of 100% was assigned to the mix that produced the best behaviour of the performance properties studied. Furthermore, in order to determine the mix that made the greatest contribution to the mechanical performance, the surface area of the different mixes evaluated was determined in proportion to the mechanical properties. In general, the mixes manufactured with AB-PFELT are seen to offer better mechanical performance at the temperatures evaluated. The results of the resistance to rutting at 50 °C indicated that the HMA24/05 and HMA14/05 mixes presented the greatest resistance to permanent deformation and moisture damage, with greater reduction in the rut depth, compared to HMA24/R and HMA14/R, respectively. We observed that in general the stiffness modulus of all the HMA mixes with AB-PFELT maintained the same behaviour at 25 °C as the HMA24/R and HMA14/R mixes. However, at the same temperature (25 °C), the HMA24/03 and HMA14/01 mixes showed better performance for indirect tensile strength. When the behaviour of the asphalt mixes at the most demanding low temperature (−10 °C) is evaluated, in general, the HMA24/03 and HMA24/03 mixes are seen to present a better response to thermal cracking; they present greater flexibility, due to their greater resistance (Fmax) and the improved bending capacity reflected in their higher d50PM value. These results indicate that the PFELT content in the asphalt binder has a variable effect on the different performance properties of the mixes evaluated. The greatest effects are observed for HMA24/05 and HMA14/03, which have the greatest areas of contribution to the mechanical performance, 97% and 88%, respectively.

## 4. Conclusions

In this article, we studied the effect of the addition of polymer fibre from ELT to two asphalt binders (CA-24 and CA-14) on the mechanical properties of the HMA at high, intermediate, and low operating temperatures. The parameters evaluated were: (1) resistance to thermal cracking; (2) stiffness modulus; (3) indirect tensile strength; and (4) resistance to permanent deformation. Based on the results obtained in the analysis of properties, related to the mechanical performance of the mixes, the following conclusions may be drawn:

1. The physical and chemical analyses showed that polymer fibre from ELT (PFELT) consists of filaments of a mean diameter 22.5 μm, with particles of rubber waste from ELT recycling adhering to the fibres. The results of the TGA curves showed that the melting point of the PFELT was 241.7 °C, with devolatilization temperature around 347 °C.
2. The best modification condition of the asphalt binder with TFELT was determined as a modification temperature of 170 °C for a period of 2 h at 350 rpm revolutions per minute.
3. In the evaluation of thermal cracking, it was found that, in general, the maximum resistance (Fmax) of the HMA mixes manufactured with AB-PFELT presented no significant differences at −10, 0, and 10 °C. However, the HMA24/03 and HMA24/05 mixes presented greater tensile strength, indicating an increase in the Fmax of around 5–14% at 10 and 0 °C. In the evaluation of bending capacity (d50PM), the HMA24 mixes manufactured with AB-PFELT showed a greater capacity to admit deformation at lower temperatures, with a significant improvement in the values of the d50PM parameter, while the HMA14 mixes manufactured with AB-PFELT showed a significant diminution in the d50PM parameter, and the mixes had a faster fracture velocity.
4. In the evaluation of the properties at intermediate temperature, the HMA mixes manufactured with AB-PFELT showed no significant effect on the stiffness modulus values at 25 °C; their performance was similar to that of HMA/R. On the other hand, it was observed that the AB-PFELT added to the asphalt mixes absorbed part of the traction forces, thus leading to a significant increase of up to 19% in the *ITS* values at 25 °C.
5. The results for the capacity of the mixes to resist permanent deformation at high temperature showed that HMA manufactured with AB-PFELT presented a significant increase in resistance to rutting, which is attributed to strengthening of the asphalt binder matrix by the addition of PFELT. This effect was most significant at higher addition rates, thus reducing the rut depth recorded for conventional mixes by up to 34% in the case of HMA24/05 and 39% in case of HMA14/05. None of the mixes evaluated presented loss of adhesion between the stone aggregates and asphalt binder (stripping), indicating that the HMA mixes modified with AB-PFELT are not susceptible to moisture damage.
6. It was determined that the greatest effects of the use of AB-PFELT were shown for the HMA24/05 and HMA14/03 mixes, with areas of contribution to mechanical performance 30% and 12% higher than the reference mixture, respectively.
7. PFELT is currently a massive residue from the ELT recycling process. This study demonstrates that PFELT can be used to produce modified AB to improve the performance of asphalt mixes. However, future studies may be conducted with other ways of incorporating PFELT into the asphalt mix to improve its performance.

## Figures and Tables

**Table 1:** Properties of the textile fibre originating from end-of-life tyres.

	Characteristic	Description	Reference
** 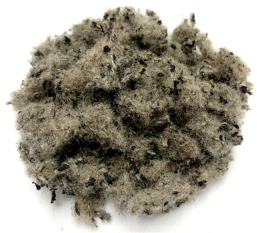 **	Colour	Dark grey	
	Form	Yarned	
	Single fibre diameter (μm)	20–40	[10,32]
	Length (mm)	1.0–2.5	[21]
	Density (gr/cm^3^)	0.07–0.287	[10]
	Thermal conductivity (W/m·K)	0.05–0.06	[10]
	Specific heat (J/kg·K)	0.12–0.20	[10]
	Diffusivity (m^2^/s)	0.30–0.50	[10]
	Tensile strength (MPa)	300–2000	[33]
	Melting point (°C)	242–272 °C	

**Table 2:** Properties of the asphalt binders used.

Tests	CA-24	CA-14	Specs. [34]
Absolute viscosity at 60 °C, 300 mm Hg (P)	2940	2226	AB24: Min 2400
			AB14: Min 1400
Penetration at 25 °C, 100 g. 5 s. (0.1 mm)	63	70	Min 40
Ductility at 25 °C, (cm)	100	>150	Min 100
Spot test hep./xyl., (% xylene)	−30	<25	Max 30
Trichloroethylene solubility (%)	99.8	99.9	Min 99
Cleveland open cup flash point (°C)	310	328	Min 232
Softening point R & B (°C)	52.2	48.6	To be reported
Penetration index	−0.1	−0.8	−1.5 a +1.0
RTFOT			
Mass loss (%). Absolute viscosity at 60 °C, 300 mm Hg (P) Ductility at 25 °C, 5 cm/min, (cm) Durability index	0.08 7860 100 2.7	0.1 6019 150 2.7	Max 0.8 To be reported Min 100 Max 4.0

**Table 3:** Physical properties of aggregates.

Tests	Results	Specifications [35]
Los Angeles abrasion loss (%)	18.4	Max. 25 (*)–35
Sodium sulphate soundness (%)	2.4	Max 12
Crushed aggregates (%)	97.3	Min. 90 (*)–70
Flakiness index (%)	0.1	Max. 10 (*)–15
Static method adhesion	>95	Min. 95
Dynamic method adhesion	>95	Min. 95
Soluble salts (%)	0.5	Max. 2 (*)–3
Sand equivalent (%)	81	Min. 50 (*)–40

(*) Wearing course.

## Data Availability

Not applicable.
